# Supplementary material for: Causal Relationship between Aging and Anorexia Nervosa: A White-Matter-Microstructure-Mediated Mendelian Randomization Analysis
Source: Biomedicines. 2024 Aug 16;12(8):1874. doi: 10.3390/biomedicines12081874 (PMC11351342; doi:10.3390/biomedicines12081874)
Supplement: Supplementary file 1 [file biomedicines-12-01874-s001.zip › Supplementary Figure/Figure S4/Singe SNP plot of MR analysis for TL to ubm-b-1979.pdf]

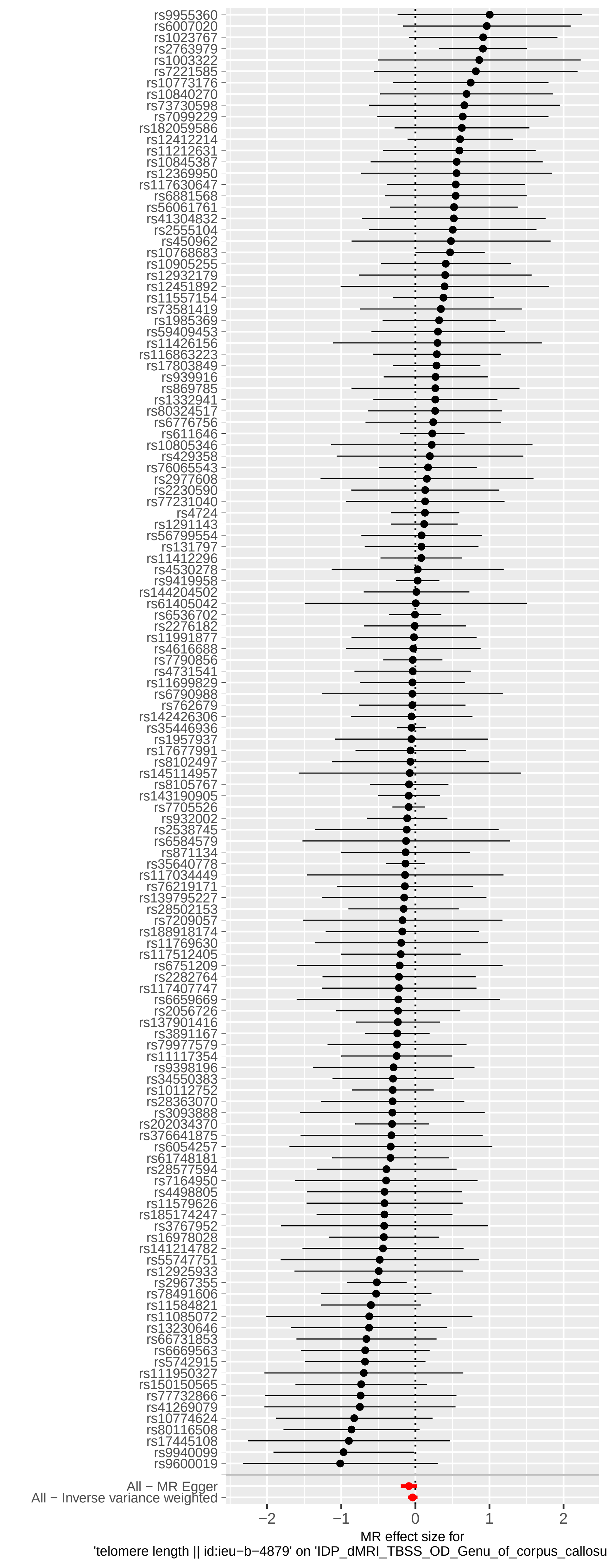
rs9955360
rs6007020
rs1023767
rs2763979
rs1003322
rs7221585
rs10773176
rs10840270
rs73730598
rs7099229
rs182059586
rs12412214
rs11212631
rs10845387
rs12369950
rs117630647
rs6881568
rs56061761
rs41304832
rs2555104
rs450962
rs10768683
rs10905255
rs12932179
rs12451892
rs11557154
rs73581419
rs1985369
rs59409453
rs11426156
rs116863223
rs17803849
rs939916
rs869785
rs1332941
rs80324517
rs6776756
rs611646
rs10805346
rs429358
rs76065543
rs2977608
rs2230590
rs77231040
rs4724
rs1291143
rs56799554
rs131797
rs11412296
rs4530278
rs9419958
rs144204502
rs61405042
rs6536702
rs2276182
rs11991877
rs4616688
rs7790856
rs4731541
rs11699829
rs6790988
rs762679
rs142426306
rs35446936
rs1957937
rs17677991
rs8102497
rs145114957
rs8105767
rs143190905
rs7705526
rs932002
rs2538745
rs6584579
rs871134
rs35640778
rs117034449
rs76219171
rs139795227
rs28502153
rs7209057
rs188918174
rs11769630
rs117512405
rs6751209
rs2282764
rs117407747
rs6659669
rs2056726
rs137901416
rs3891167
rs79977579
rs11117354
rs9398196
rs34550383
rs10112752
rs28363070
rs3093888
rs202034370
rs376641875
rs6054257
rs61748181
rs28577594
rs7164950
rs4498805
rs11579626
rs185174247
rs3767952
rs16978028
rs141214782
rs55747751
rs12925933
rs2967355
rs78491606
rs11584821
rs11085072
rs13230646
rs66731853
rs6669563
rs5742915
rs111950327
rs150150565
rs77732866
rs41269079
rs10774624
rs80116508
rs17445108
rs9940099
rs9600019
All − MR Egger
All − Inverse variance weighted
−2
−1
0
1
2
MR effect size for
'telomere length || id:ieu−b−4879' on 'IDP_dMRI_TBSS_OD_Genu_of_corpus_callosu
